# The molecular mechanism for inhibiting the growth of nasopharyngeal carcinoma cells using polymethoxyflavonoids purified from pericarp of *Citrus reticulata* ‘Chachi’ via HSCCC

**DOI:** 10.3389/fphar.2023.1096001

**Published:** 2023-04-27

**Authors:** Wanling Yang, Yiyao Liang, Yujie Liu, Baizhong Chen, Kanghui Wang, Xiaojing Chen, Zhiqian Yu, Depo Yang, Yi Cai, Guodong Zheng

**Affiliations:** ^1^ Guangzhou Municipal and Guangdong Provincial Key Laboratory of Molecular Target and Clinical Pharmacology, The NMPA and State Key Laboratory of Respiratory Disease, School of Pharmaceutical Sciences and the Fifth Affiliated Hospital, Guangzhou Medical University, Guangzhou, China; ^2^ Guangdong Xinbaotang Biological Technology Co., Ltd., Jiangmen, China; ^3^ School of Pharmaceutical Sciences, Sun Yat-sen University, Guangzhou, China

**Keywords:** *Citrus reticulata* ‘Chachi’ pericarp, high-speed counter-current chromatography, polymethoxyflavonoids, proliferation, AMPK signaling pathway

## Abstract

Polymethoxyflavonoids (PMFs), the main bioactive compounds naturally occurring in the pericarp of *Citrus reticulata* ‘Chachi’ (CRCP), possess significant antitumor action. However, the action of PMFs in nasopharyngeal carcinoma (NPC) is currently unknown. The present research study was conducted to investigate the inhibitory mechanisms of PMFs from CRCP on NPC growth *in vivo* and *in vitro*. In our research, we used high-speed counter-current chromatography (HSCCC) to separate four PMFs (nobiletin (NOB), 3,5,6,7,8,3′,4′-heptamethoxyflavone (HMF), tangeretin (TGN), and 5-hydroxy-6,7,8,3′,4′-pentamethoxyflavone (5-HPMF)) from CRCP. CCK-8 assay was used to preliminarily screen cell viability following exposure to the four PMFs. Colony formation, Hoechst-33258 staining, transwell, and wound scratch assays were performed to assess the anti-proliferation, invasion, migration, and apoptosis-inducing effects of HMF on NPC cells. NPC tumors in xenograft tumor transplantation experiments were also established to explore the effect of HMF (100 and 150 mg/kg/day) on NPC. The histopathological changes in the treated rats were observed by H&E staining and Ki-67 detection by immunohistochemical techniques. The expressions of P70S6K, p-P70S6K, S6, p-S6, COX-2, p53, and p-p53 were measured by Western blot. The four PMFs were obtained with high purity (>95.0%). The results of the preliminary screening by CCK-8 assay suggested that HMF had the strongest inhibitory effect on NPC cell growth. The results of the colony formation, Hoechst-33258 staining, transwell, and wound scratch assays indicated that HMF had significant anti-proliferation, invasion, migration, and apoptosis-inducing ability in NPC cells. Moreover, HMF suppressed NPC tumor growth in xenograft tumor transplantation experiments. Further investigation suggested that HMF regulated NPC cells proliferation, apoptosis, migration, and invasion by activating AMPK-dependent signaling pathways. In conclusion, HMF-induced AMPK activation inhibited NPC cell growth, invasion, and metastatic potency by downregulating the activation of the mTOR signaling pathway and COX-2 protein levels, as well as enhancing the p53 phosphorylation level. Our study provides a crucial experimental basis for the clinical treatment of NPC, as well as the development and utilization of PMFs from CRCP.

## 1 Introduction

Nasopharyngeal carcinoma (NPC), emerging from the nasopharyngeal epithelium, is especially common in East and Southeast Asia (Lee et al., 2019). The delayed treatment and worse prognosis occur mainly due to the hidden early clinical symptoms. Intensity-modulated radiotherapy (IMRT) is considered ideal for the local treatment of NPC for its dose protection on organs around the nasopharynx, whereas distant metastases also occur in some patients treated with IMRT; these tumor cells develop resistance to chemotherapy and radiotherapy in the later period of treatment (Perri et al., 2019). Collectively, metastasis, relapse, resistance to chemotherapy, and side effects are urgent clinical problems in NPC treatment. Hence, developing naturally efficient and low-toxicity drugs as NPC therapeutics is of urgent need (Wang C Y et al., 2020).

Based on the Chinese Pharmacopoeia (2020 edition), *Citrus* *reticulata* ‘Chachi’, a major cultivar of *Citrus reticulata* Blanco, is planted and harvested in Xinhui region, Guangdong Province in China (Pharmacopoeia, 2020). Polymethoxyflavonoids (PMFs) naturally occurring in citrus have been identified as the main bioactive non-volatile constituents in the pericarpium of *Citrus reticulata* ‘Chachi’ (CRCP), mainly including nobiletin (NOB), 3,5,6,7,8,3′,4′-heptamethoxyflavone (HMF), tangeretin (TGN), and 5-hydroxy-6,7,8,3′,4′-pentamethoxyflavone (5-HPMF) (Kong et al., 2020; Nair et al., 2018). Emerging studies have reported that PMFs strongly inhibit tumor cell proliferation (Shi et al., 2013). For instance, TGN has proliferation inhibitory and apoptosis induction effects in three gastric cancer cell lines (AGS, SGC-7901, and BGC-823) (Wang Y et al., 2020). Moreover, 5-HPMF led to apoptosis induction and cell cycle block in HCT116 colon cancer cells (Qiu et al., 2011). We also previously demonstrated the growth inhibition and apoptosis induction of nobiletin in NPC cells (Zheng et al., 2019). Nevertheless, the mechanism of action of PMFs in CRCP on NPC is unknown and worth exploring in further studies.

AMP-activated protein kinase (AMPK) is capable of regulating cell metabolism in eukaryotic cells and is crucial for maintaining energy homeostasis in the human body (Tokunaga et al., 2019). AMPK activation elicited the inhibition of malignant tumor occurrence and development by inhibiting tumor cell proliferation, migration, and invasion and inducing apoptosis. For instance, the AMPK activator (Acadesine, AICAR) inhibited the proliferation and induced apoptosis in HepG2/C3A, Huh-7, and SK-HEP-1 hepatoma cells (Ferretti et al., 2016). In contrast, the effect of apoptosis-inducing ginsenoside metabolites on non-small cell lung cancer cells A549 and H1975 could be reversed by AMPK inhibition (Li et al., 2019). Moreover, metformin-induced AMPK activation inhibited the proliferation and induced apoptosis of NPC C666-1 cells, suggesting that AMPK is a potential target for NPC therapy (Zhao et al., 2011).

In the present study, the high-speed counter-current chromatography (HSCCC) method was established for the isolation and preparation of four PMF monomeric components from CRCP for the first time. Cell counting kit-8 (CCK-8) assays were used to further screen the four monomeric compounds for their proliferation-inhibiting effects on NPC cells. Moreover, colony formation, Hoechst-33258 staining, transwell, and wound scratch assays were performed to examine the anti-proliferation, invasion, migration, and apoptosis-inducing abilities following HMF treatment of NPC cells. The xenograft NPC-tumor transplantation experiments were constructed *in vivo*, and H&E staining and Ki-67 detection by immunohistochemical technique were performed to observe histopathological changes to investigate the anti-NPC effect of HMF (100 and 150 mg/kg/day). In addition, the molecular mechanism of the PMF components in regulating the apoptotic, migratory, and invasive effects of NPC cells in combination with the AMPK-mTOR signaling pathway was explored.

## 2 Materials and methods

### 2.1 Herbal medicine

The *Citrus reticulata* “Chachi” pericarp (CRCP), purchased from Xinbaotang Biological Technology Co., Ltd. (Jiangmen, Guangdong, China) in January 2018, was identified by Prof. Guodong Zheng. Meanwhile, the CRCP samples were stored at the Pharmacognosy Laboratory of Guangzhou Medical University.

### 2.2 Chemicals and reagents

Chromatographical acetonitrile was bought from Thermo Fisher Scientific (China). Analytical-grade petroleum ether (PE), methanol, and ethyl acetate were bought from Honeywell (USA). Ultrapure water was obtained from a Milli-Q system (Millipore, USA). The other chemicals were analysis-grade chemicals.

RPMI 1640 medium and fetal bovine serum (FBS) were purchased from Gibco (Logan, UT, USA). Cell counting kit 8 (CCK-8) was bought from DOJINDO (Japan). Antibodies (including β-actin, p-AMPK, AMPK, p-S6, S6, p-P70S6K, P70S6K, p-p53, p53, and COX-2) were purchased from Cell Signaling Technology (USA); Compound C (an AMPK inhibitor) was bought from Santa Cruz Biotech (USA). Matrix basement membrane was purchased from Corning Co., Ltd. (USA). The apoptosis-Hoechst Staining Kit was purchased from Beyotime Biotechnology Co., Ltd. (Shanghai, China). Crystal violet reagent was purchased from Damao (Tianjin, China). Ki-67 was purchased from Wuhan Servicebio Technology Co., Ltd. The four PMFs obtained from CRCP were used in DMSO and were stored at −20°C and diluted for use.

### 2.3 Simultaneous purification of four PMFs from CRCP by HSCCC

#### 2.3.1 Enrichment of PMFs from CRCP

The CRCP was pulverized into powder and passed through a 20-mesh sieve (1 kg). The powder samples were then extracted four times by PE solvent heat reflux extraction. The parameters (liquid to solid ratio) were 4:1, 3:1, 3:1, and 3:1, with an extraction time of 1 h, respectively. The aforementioned four extracting solutions were merged for rotatory evaporation until deposition occurred. The residues were weighed accurately after drying and kept at 4°C.

The HPLC-PDA method for the aforementioned PE extracts was performed on the Dikma Diamonsil C_18_ column (250 mm × 4.6 mm i.d., 5 μm). The elution system was composed of 50% water (phase A) and 50% acetonitrile (phase B). The flow velocity was 1 mL·min^-1^, and the injection sample load was 10 μL. All samples were verified at 330 nm and 25°C after filtering through a 0.22-μm membrane.

#### 2.3.2 Purification and identification of four PMFs from CRCP

A solvent system including petroleum ether–ethyl acetate–methanol–water (1:0.8:0.9:1, v/v/v/v) was chosen for further HSCCC purification of PMFs by comparing the partition coefficient (K = A_1_/A_2_) values by the peak areas of the PMFs by HPLC-PDA (A_1_ and A_2_) and the peak shape in HSCCC. In each HSCCC separation, the column was first filled with the upper (stationary) phase, and then the rotary was set at 1,000 rpm. The other apparatus was set as follows: column temperature 35°C and detection wavelength 330 nm. The bottom (mobile) phase was then pumped into the column at a flow rate of 2.0 mL·min^-1^. When the two phases reached hydrodynamic equilibrium, the PE extract deposit (200 mg) was dissolved in the bottom phase solution (10 mL) for injection. All effluent was detected at a wavelength of 330 nm, and sample fractions (3 mL of each test tube) were gathered for further analysis and other research.

The sample fractions were dissolved in trichloro-deuterio-methane to identify compounds by ^1^H-NMR, ^13^C-NMR, and ESI-MS. The purity analyses were performed by the normalization method of the peak area. The HPLC-PDA method was performed as described in Section 2.3.1.

### 2.4 Cell culture

CNE-2 and 5-8F cells were cultured in RPMI 1640 with certain percentages of FBS in an incubator (air to CO_2_ ratio 95:5) at 37°C. Replacement of the medium was performed every other day. As the cells reached 80%–90% confluency, trypsin was added to digest cells for the proper time, and then the cells were subcultured. Before treatment with PMFs purified from CRCP, the cells were incubated in RPMI 1640 containing free FBS for 12 h.

### 2.5 Cell viability

CNE-2 and 5-8F cells were cultured in 96-well cell plates (5×10^3^/well). Next, 100 μL of RPMI 1640 medium (10% FBS) was added to 96-well cell plates, and the plates were incubated in 5% CO_2_, at 37°C for 24 h. Replacement of the FBS-free medium was carried out for 12 h on cells before treatment with HMF. Each well was supplied with CCK-8 liquid (10 μL) for 1 h. The absorbance measurement was performed on a Multi-Volume Spectrophotometer system (BioTek Instruments, Inc., USA) at 450nm.

### 2.6 Colony formation assay

CNE-2 and 5-8F cells were inoculated into six-well cell plates (800/well). RPMI 1640 medium containing 10% FBS (2 mL) was added and incubated in a cell incubator (5% CO_2_, 37°C) for 24 h to allow the cells to completely adhere to the wall. Then, the cells were incubated with media containing HMF at different concentrations (10 μM, 25 μM, and 50 μM), respectively. The incubation medium was changed every 2 days while maintaining the original drug concentrations. After 14 days, colonies with >50 cells stained with crystal violet (0.5%) at room temperature, were counted on a stereomicroscope after 1 h. Relative clone-forming efficiency (%) = (number of cell colonies with more than 50 cells in the administration group/ number of cell colonies with cells more critical than 50 in the control group) × 100%.

### 2.7 Hoechst-33258 staining

CNE-2 and 5-8F cells were plated at 9×10^4^/well into 12-well plates supplemented with 1 mL of medium (10% FBS) and incubated at 37°C overnight. Next, 4% paraformaldehyde (500 μL/well) was used to fix the cells at room temperature for 10 min after treatment with HMF (10 μM, 25 μM, 50 μM) for 24 h. Then, 500 μL of Hoechst-33258 (Beyotime, China) was added to stain the cells for 5 min at room temperature in the dark, washing twice with PBS. A liquor of 50 μL anti-fluorescence sealing was added to prevent fluorescence quenching. A fluorescence microscope was used to observe and image apoptotic morphological features (chromatin condensation and nuclear fragmentation) (20×).

### 2.8 Wound scratch healing

CNE-2 and 5-8F cells were inoculated into six-well plates (3×10^5^/well) supplemented with medium for 24 h. Semblable straight scratches were made on monolayers in each well with sterile pipette tips, which were then washed twice with 1 mL PBS. An inverted microscope (10×) was used to take representative pictures at 0 h. After that, the wounds were treated with HMF (10 μM, 25 μM, and 50 μM) for 24 h, respectively, and then the same scratch position at 0 h was photographed. ImageJ software was used to measure and analyze the areas between the edges of the wounds. Mobility (%) = (0 h blank area ˗ 24 h blank area)/0 h blank area × 100%.

### 2.9 Transwell migration assay

Matrix glue (50 mg/L) was diluted with RPMI 1640 medium without serum (v/v, 1:8). A matrix glue dilution (45 μL) was appended to the upper transwell chamber. An incubator (5% CO_2_) at 37°C was used to incubate for 2 h. The excess medium was removed after forming. CNE-2 and 5-8F cell suspensions incubated with HMF (10 μM, 25 μM, 50 μM) were spread in Matrigel plate wells with serum-free RPMI 1640 medium (2.5×10^6^ cells/well). The lower well was supplied with 600 μL of complete medium (RPMI 1640 and 10% FBS) to stimulate cell migration. The cells were incubated for 24 h and those that had not migrated through the Matrigel plate wells were wiped off with swabs. A total of 1 mL of paraformaldehyde (4%) was applied to fix the cells in the bottom chamber for 30 min. Pictures of the cells were taken under the microscope (10×) and counted in ImageJ software after staining with 1 mL of 1% crystal violet at room temperature for 1 h.

### 2.10 Western blot analysis

The expression levels of AMPK-dependent signaling pathway proteins (P70S6K, p-P70S6K, S6, p-S6, COX-2, p53, and p-p53) were examined by Western blot analysis. Cells (CNE-2 and 5-8F) were inoculated into six-well plates (2×10^6^/well), incubated with HMF (10 μM, 25 μM, and 50 μM) for 48 h, and then lysed with RIPA buffer with an added inhibitor cocktail of protease and phosphatase. The BCA assay method was used to measure protein concentrations. Appropriate quantities of protein samples (20 μg) were subjected to polyacrylamide gel electrophoresis and transferred to PVDF membranes. The membranes were then incubated with 5% skim milk for 1 h at room temperature and then incubated with the primary antibodies (p-AMPK, AMPK, p-P70S6K, P70S6K, p-S6, S6, COX-2, p-p53, and p53) at 4°C overnight before washing three times with TBST. Then, the membranes were incubated with an appropriate peroxidase-conjugated secondary antibody (1:1,500) at room temperature for 1 h. Finally, enhancement of the immunoreactivity signals was detected by a chemiluminescence system. The relative density of the target bands was statistically analyzed using Quantity One analysis software. β-Actin was used for protein level normalization.

### 2.11 Construction of the transplanted tumor model *in vivo*

#### 2.11.1 Construction of nude mice tumor-bearing model

Thirty male nude mice (6 weeks old, 18–22 g) were bought from Guangzhou Jingwei Biological Co., Ltd. (Guangdong, China). The environment is under specific pathogen-free conditions between 22°C and 28°C and give free access to food and water. CNE-2 (1.5×10^6^ cells) were suspended in PBS and Matrigel matrix solution (1:1, v/v) and subcutaneously injected into the axilla of the right forelimb of the nude mice. When the mean tumor volume reached approximately 100 mm^3^, the mice were randomly and equally divided into three groups (n = 10), and administered oral HMF (100 and 150 mg/kg/day) for 14 days, respectively. At the same time, the normal control (NC) group was administered 0.5% CMC-Na. The weights of the mice were recorded daily, and the tumor volume was weighed on the scales and measured with a ruler. The curve of changes was plotted. The tumor volume was calculated as the long diameter length × short diameter^2^ × 0.5.

#### 2.11.2 Hematoxylin and eosin (H&E) staining

H&E staining was performed as previously described (Zhang et al., 2018). The nude-mouse transplanted tumor was removed and weighed. Then, the tumor tissue was stored in a 4% paraformaldehyde solution to maintain cell morphology and structure for >24 h. The tumor tissues were then embedded in paraffin, cut into slices (8 μm), and stained with H&E. The stained slices were scanned under a light microscope (Olympus, Japan) to evaluate tumor damage.

#### 2.11.3 Immunohistochemistry (IHC)

The tissue sections were first dewaxed and hydrated. Antigen retrieval of Ki-67 was performed with a steamer with citrate buffer, pH 7, for 32 min. The tissue sections were then incubated with primary polyclonal mouse monoclonal antibodies against Ki-67. Biotinylated goat anti-mouse IgG and IgM (200 mg·ml^−1^) were used as secondary antibodies. The I-ViewTM DAB Detection Kit was used for endogenous peroxidase blocking, followed by an ethanol gradient for rehydration. Neutral gum was used to seal the slices. Finally, the slices were inspected via a microscope. Ki-67 cells were considered positive when the cells were stained brownish yellow.

### 2.12 Statistical analysis

All experimental data were described as means ± standard deviation (mean ± SD), and each experiment was repeated three times (n = 3). Data analyses were performed on one-way ANOVA LSD statistics using SPSS 16.0 software. The significance of the differences was indicated as *p < 0.05 and **p < 0.01.

## 3 Results

### 3.1 Four PMFs purified via HSCCC from PE deposits in CRCP

**FIGURE 1:**
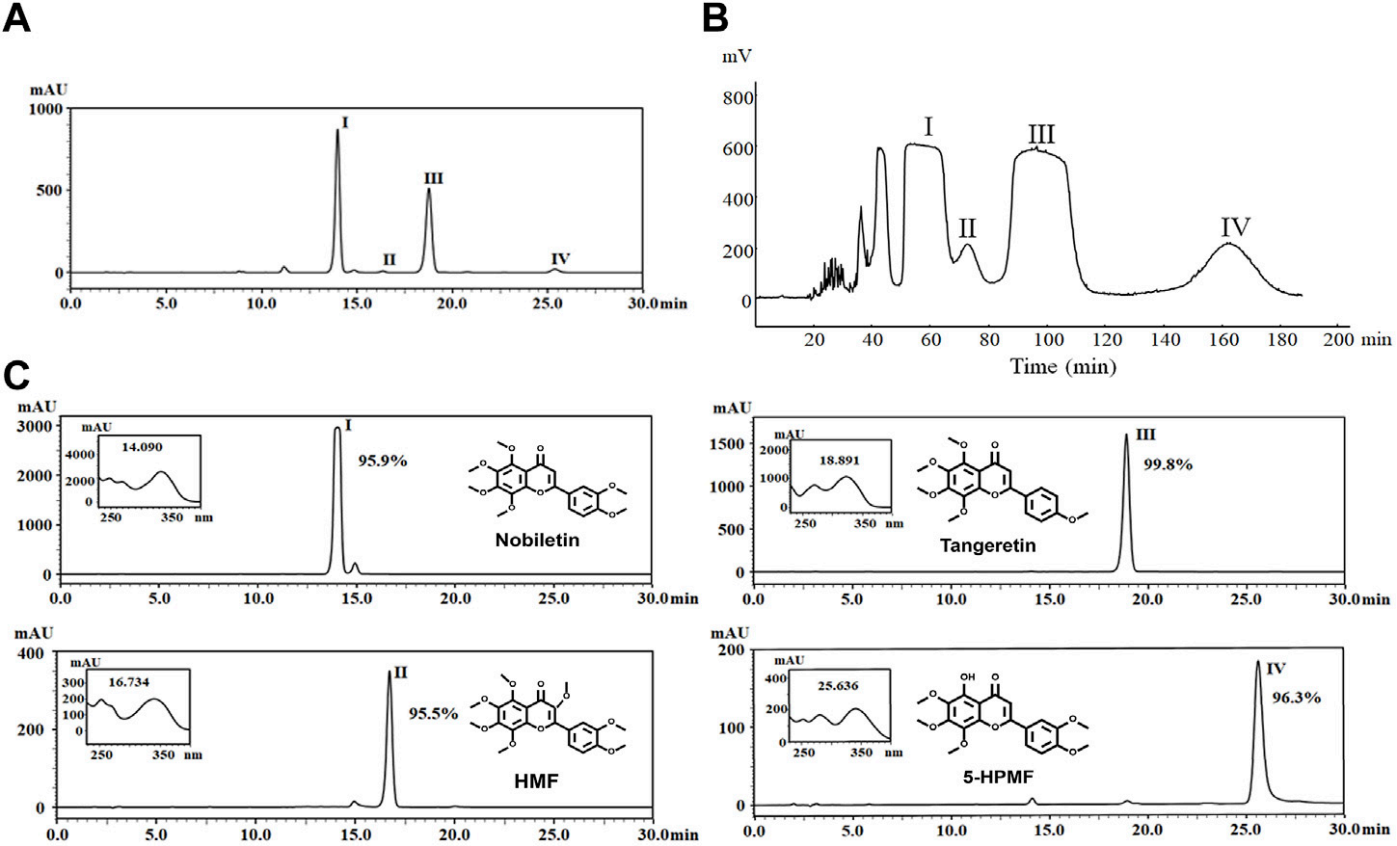
**(A)** HPLC chromatogram of nobiletin (I), HMF (II), tangeretin (III), and 5-HPMF (IV) purified from a PE deposit of CRCP at 330 nm. **(B)** Chromatogram of HSCCC separation for components (I–IV) at 330 nm and a flow rate of 2 mL·min^–1^. Fractions (I–Ⅳ) were collected from the effluent. **(C)** Identification and purity analysis of fractions (I–IV) by ESI-MS, ^1^H-NMR, ^13^C-NMR, and HPLC-PDA.

Approximately 1 mg of petroleum ether extract enriched from CRCP was dissolved in 10 mL of methanol, from which four compounds (I–Ⅳ) were detected by the HPLC-PDA method (Figure 1A). These included 325 mg of Compound I, 67 mg of Compound II, 249 mg of Compound III, and 90 mg of Compound Ⅳ, which were obtained after separation by HSCCC (Figure 1B). The purity examination was analyzed by HPLC-PDA and calculated using the peak area normalization method, which suggested that these compounds reached 95.0% purity (95.9%, 95.5%, 99.8%, and 96.3% for Compounds I–IV), respectively. ESI-MS, ^1^H-NMR, and ^13^C-NMR analyses were further performed to identify the structures of the four purified PMFs (5-HPMF, TGN, NOB, and HMF) (Figure 1C).

### 3.2 Effects of the four PMFs on the viability of 5-8F and CNE-2 cell lines

**FIGURE 2:**
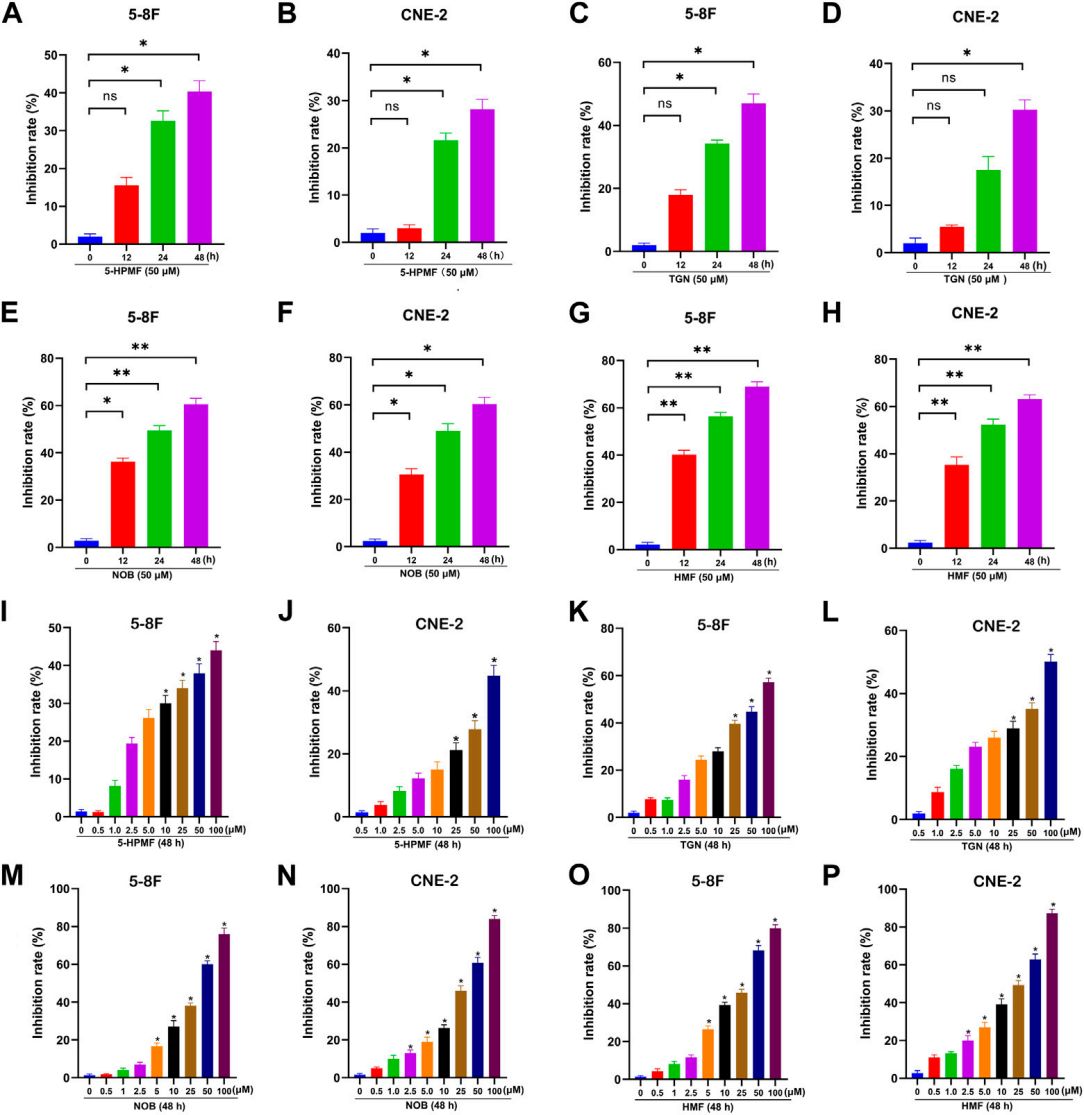
Anti-proliferation effects of the four PMFs (5-HPMF, TGN, NOB, and HMF) in human nasopharyngeal carcinoma cells. **(A–H)** 5-8F and CNE-2 cell lines were incubated with the four PMFs (50 μM) for 12 h, 24 h, and 48 h, respectively. **(I–P)** 5-8F and CNE-2 cell lines were incubated with different concentrations (0, 0.5, 1, 2.5, 5, 10, 25, 50, and 100 μM) of the four PMFs for 48 h. Cell viability was examined by CCK-8 assay. All data were described as the mean ± SD of three independent and repeated experiments. Compared to the control group (0 μM): *p < 0.05; **p < 0.01; ns, not significant.

To evaluate the anti-NPC effects of the four PMFs (5-HPMF, TGN, NOB, and HMF), the cell viability of two NPC cell lines treated with various concentrations of monomeric PMFs (0, 0.5, 1, 2.5, 5, 10, 25, 50, and 100 μM) for 48 h and treated with monomeric PMFs (50 μM) for 12 h, 24 h, and 48 h, respectively, was analyzed by CCK-8 assay. As indicated in Figure 2, the four PMFs (5-HPMF, TGN, NOB, and HMF) could inhibit the proliferation of 5-8F and CNE-2 cells lines in time- and dose-dependent manners (*p < 0.05 and **p < 0.01). The IC_50_ values of 5-HPMF and TGN in the two NPC cell lines exceeded >50 μM. The IC_50_ values of NOB were 36.579 ± 0.659 μM (CNE-2) and 37.256 ± 0.365 μM (5-8F cells), respectively. HMF showed the strongest cytotoxicity, with IC_50_ values of 36.273 ± 0.502 μM (CNE-2) and 31.786 ± 0.841 μM (5-8F cells). Thus, HMF was selected for further research on its anti-NPC effects.

### 3.3 HMF inhibits proliferation and induces apoptosis in 5-8F and CNE-2 cell lines

**FIGURE 3:**
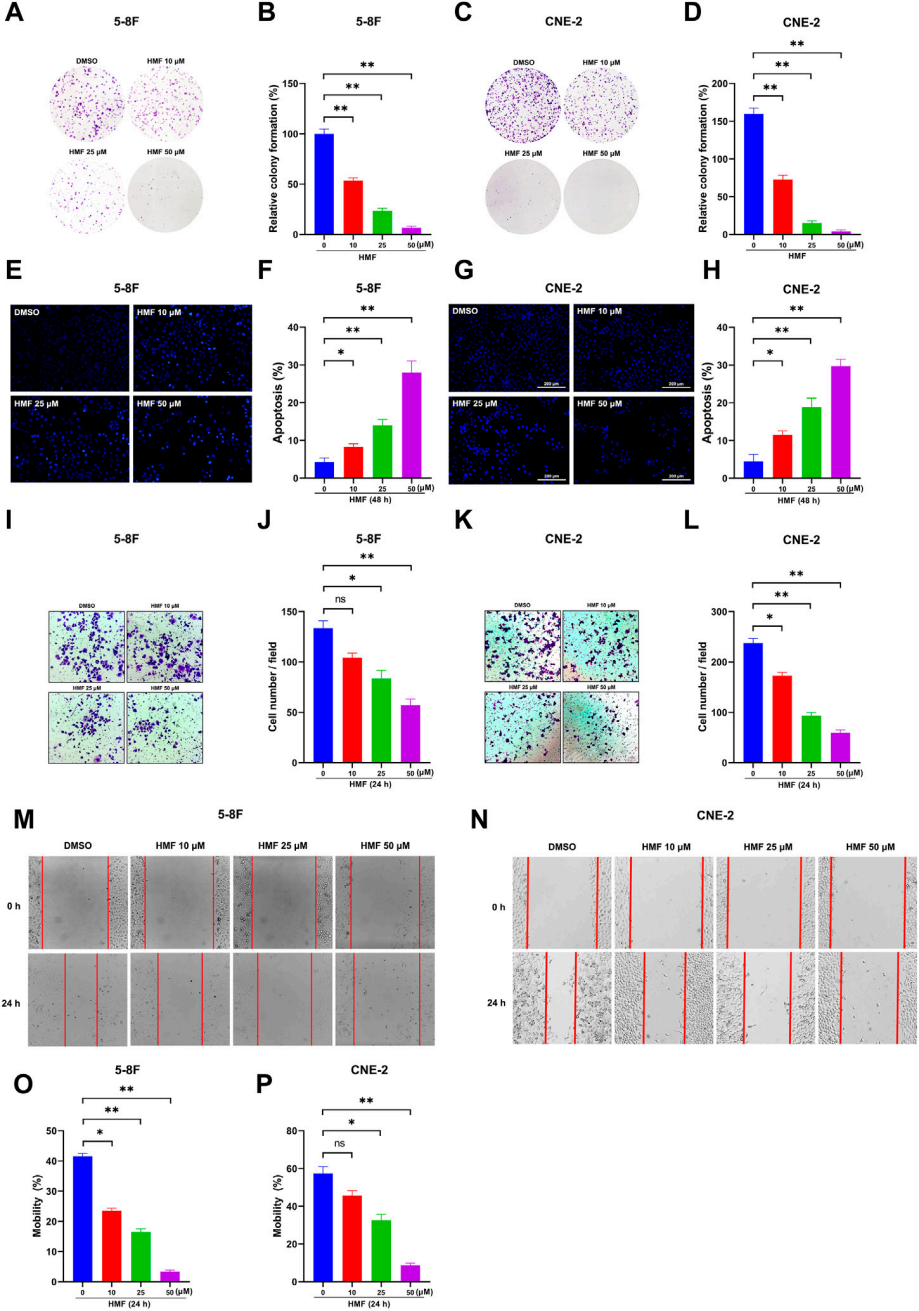
HMF induces NPC cell apoptosis and inhibits migration and invasion. **(A,C)** The indicated numbers of cells were incubated in six-well dishes and treated with HMF (0, 10, 25, and 50 μM). After 7 days, the colonies were stained with 0.5% crystal violet. **(B,D)** The number of colonies containing ≥50 cells and the relative colony formation were counted under a microscope to quantify the proliferation. **(E,G)** 5-8F and CNE-2 cells were stained with Hoechst-33258 after incubation with different concentrations (0, 10, 25, and 50 μM) of HMF. The morphological changes were analyzed using a fluorescence microscope (20×). **(F,H)** Apoptosis (%) of 5-8F and CNE-2 cells treated with HMF (0, 10, 25, and 50 μM) for 24 h. **(I,K)** Representative images of transwell-migration assays of 5-8F and CNE-2 cells treated with HMF (0, 10, 25, and 50 μM) for 24 h (10×). **(J,L)** Quantification of cell migration expressed by cell counting. **(M,N)** Representative images of scratch assays performed in 5-8F and CNE-2 cells with HMF treatment (0, 10, 25, and 50 μM) for 24 h (10×). **(O,P)** Mobility (%) was measured 24 h after the cells were scratched. All data are described as the mean ± SD of three independent and repeated experiments. Compared to the control group (0 μM): *p < 0.05; **p < 0.01; ns, not significant.

Based on the aforementioned results, we focused on the anti-NPC effects of HMF in further studies. As Figures 3A–D show, the number of cell colonies containing ≥50 cells and the relative colony formation were significantly decreased in cells treated with HMF (10, 25, 50 μM) compared to the control group after 7 days. These findings further indicated that HMF suppressed 5-8F and CNE-2 cell proliferation. To simultaneously explore the anti-apoptotic effects of HMF on NPC cell lines, we performed Hoechst-33258 staining and examined the samples on a fluorescence microscope. The morphological changes of 5-8F and CNE-2 cell lines treated with HMF (10, 25, and 50 μM) were observed on a fluorescence microscope (20×). Compared with the control group, 5-8F and CNE-2 cells in the HMF group showed condensation of nuclear chromatin staining, atomic shrinkage, and other apoptosis phenomena. The apoptosis rate of the HMF group was remarkably higher than that in the control group (*p < 0.05 and **p < 0.01). Among them, the apoptotic effect of high-dose HMF (50 μM) groups was the strongest (Figures 3E–H). These results indicated that HMF significantly induced growth inhibition and apoptosis in NPC cell lines (5-8F and CNE-2).

### 3.4 HMF inhibits invasion and migration of 5-8F and CNE-2 cell lines

To determine the effect of HMF on the migration and invasion of CNE-2 and 5-8F cells, wound-healing and transwell-migration assays were performed. As shown in Figures 3I–L, the HMF dose dependently inhibited the invasion of CNE-2 and 5-8F cells. The wound healing levels calculated at two time points (0 h and 24 h) showed remarkably weaker cell migration ability in HMF groups (10, 25, and 50 μM) compared to the control groups in the two NPC cell lines (Figures 3M–P) (*p < 0.05, **p < 0.01). These results demonstrated that HMF dose-dependently inhibited the invasion and migration of NPC cells (5-8F and CNE-2).

### 3.5 HMF regulates the AMPK-dependent signaling pathway in NPC cells

**FIGURE 4:**
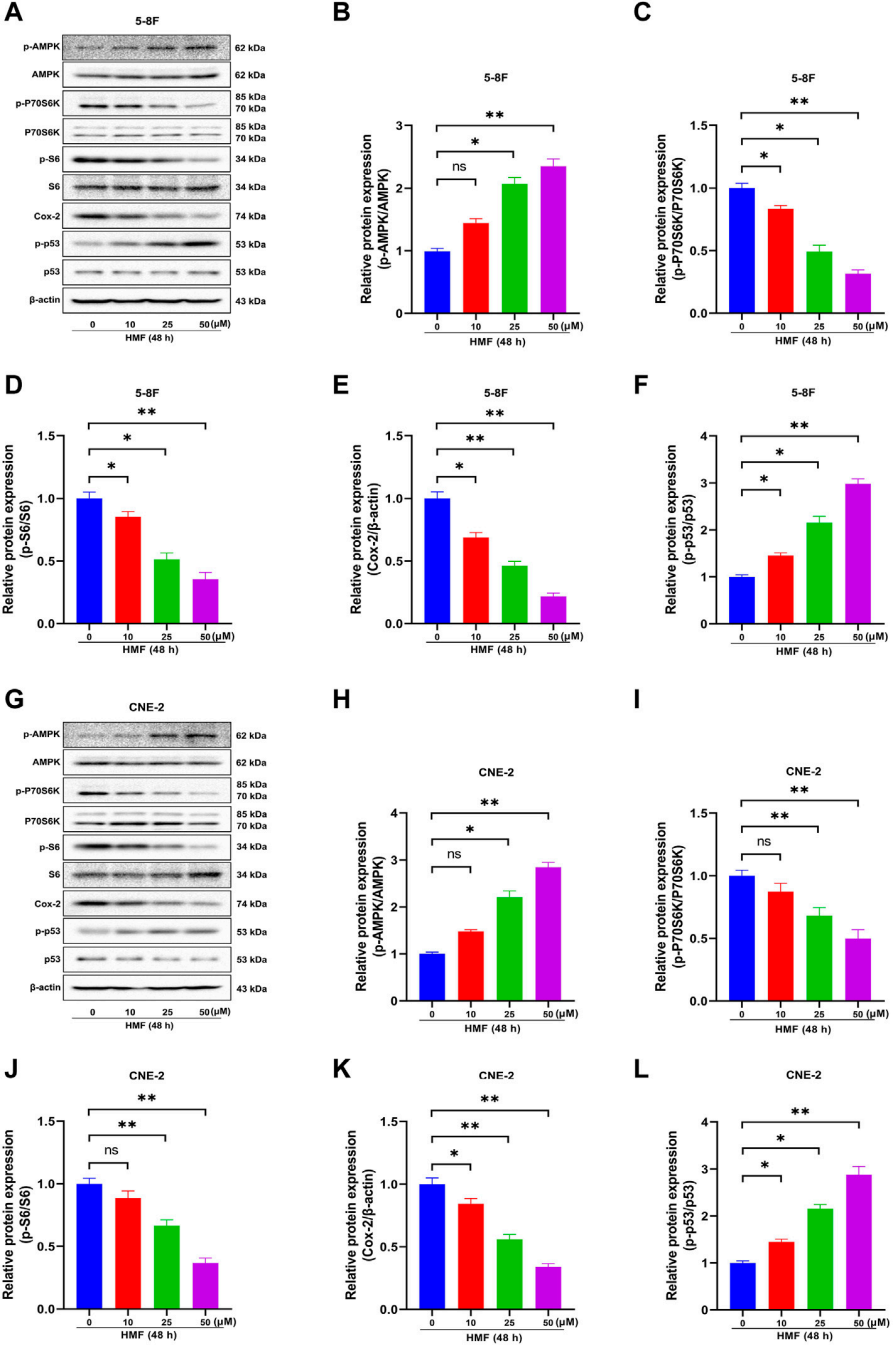
Effect of HMF on 5-8F and CNE-2 human nasopharyngeal carcinoma cells through the AMPK signaling pathway. **(A,G)** 5-8F and CNE-2 cell lysates treated with HMF (0, 10, 25, and 50 μM) assessed by Western blot analysis with antibodies against p-AMPK, AMPK, p-P70S6K, P70S6K, p-S6, S6, COX-2, p-p53, and p53. β-Actin served as the loading control. **(B–D)** and **(H–J)** Quantification of phosphorylation expression levels of key AMPK-mTOR pathway molecules (p-AMPK/AMPK, p-P70S6K/P70S6K, and p-S6/S6) in two NPC cells lines. **(E,K)** Quantification of COX-2 expression levels. **(F,L)** Quantification of p53 phosphorylation levels. All data are described as the mean ± SD of three independent and repeated experiments. Compared to the control group (0 μM): *p < 0.05, **p < 0.01; ns, no significance.

AMPK is a crucial regulator of cellular energy metabolism. AMPK activation participates in the body’s metabolic and energy regulation by regulating multiple related pathways, accounting for apoptosis and proliferation inhibition in cancer cells. The protein levels of p-AMPK, AMPK, p-P70S6K, P70S6K, p-S6, S6, COX-2, p-p53, and p53 in two NPC cell lines were detected by Western blot to explore the effects of the AMPK signal cascade on HMF-induced NPC cell apoptosis and proliferation inhibition. As shown in Figures 4A–F, in 5-8F cells, the HMF treatment group (25 and 50 μM) showed significantly upregulated protein levels of phosphorylated AMPK (p-AMPK/AMPK). At the same time, phosphorylated P70S6K and S6 (p-P70S6K/P70S6K and p-S6/S6) levels were dose-dependently and significantly reduced. These results suggested that HMF could inhibit the AMPK-mTOR signaling pathway in 5-8F cells. Moreover, the protein levels of COX-2 were downregulated in the HMF group compared to the control group, suggesting that HMF induced the activation of the AMPK/COX-2 signaling pathway in both cell lines. Meanwhile, compared to the control group, the HMF treatment group showed significantly upregulated p-p53/p53, indicating that HMF inhibited NPC cell growth and proliferation through the AMPK/p53 signaling pathway (*p < 0.05 and **p < 0.01). We also showed that HMF inhibited the AMPK-dependent pathway in NPC CNE-2 cells (Figures 4G–L).

### 3.6 HMF inhibits proliferation and induces apoptosis by regulating the AMPK-dependent signal pathway in NPC cells

**FIGURE 5:**
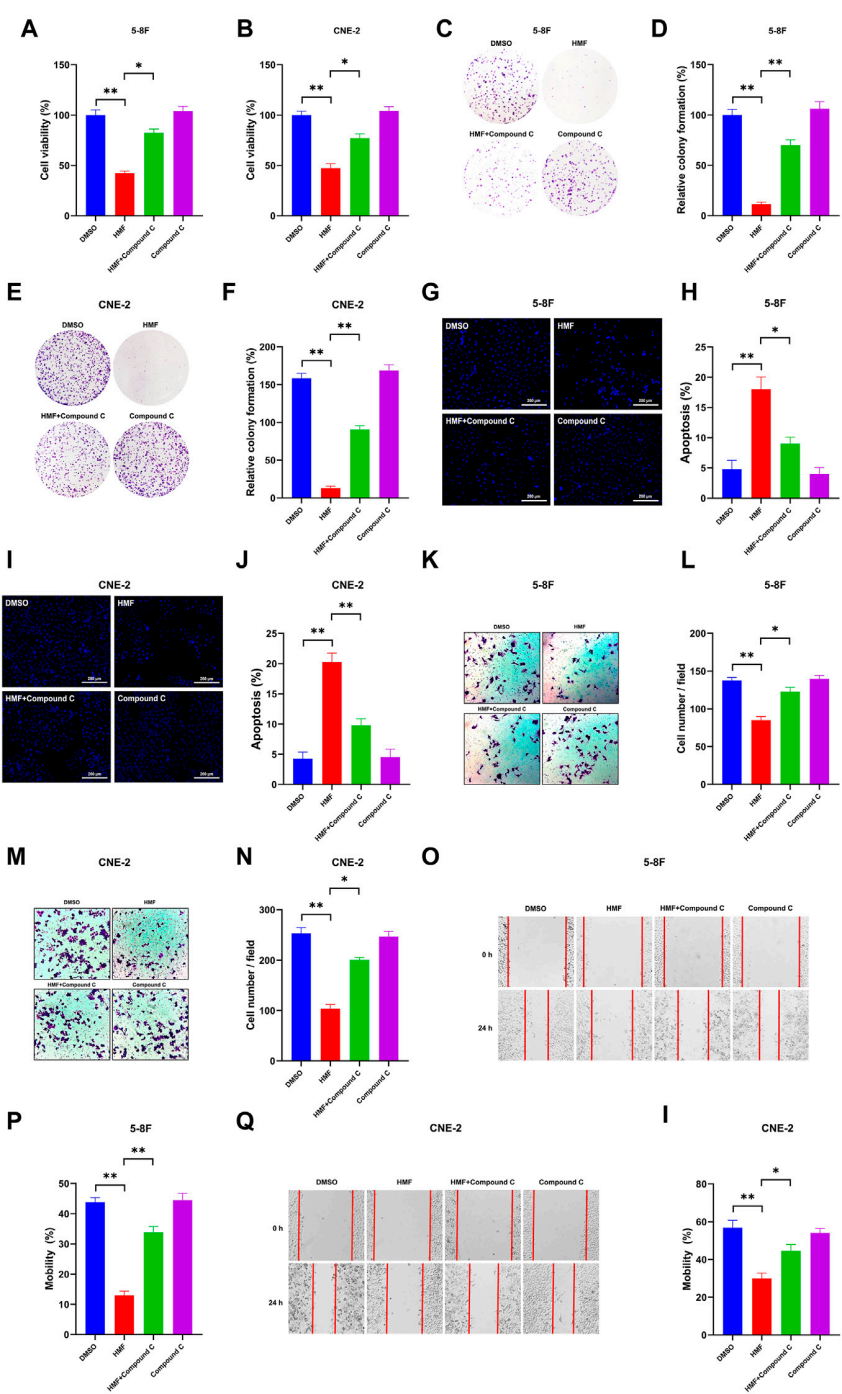
Effects of AMPK on proliferation, apoptosis, and metastasis through regulation of the AMPK-dependent signal pathway in two NPC cell lines. After high-dose HMF (50 μM) co-treated with Compound C (AMPK inhibitor) for 24 h. **(A,B)** The cell viability was determined by CCK-8 assay. **(C–F)** The colonies were stained with 0.5% crystal violet, and the colony numbers were quantified. **(G,I)** Assessment of the morphological changes in each treatment group using a fluorescence microscope (20×). **(H,J)** Apoptosis (%) in each group. **(K–N)** Representative images of transwell migration assays (10×) and quantification of cell migration expressed by cell counting. **(O–I)** Representative images of the scratch assay (10×) and mobility (%)24 h after the cells were scratched. All data are described as the mean ± SD of three independent and repeated experiments. Compared to the control group (0 μM): *p < 0.05; **p < 0.01; ns, not significant.

To further confirm whether the AMPK-dependent pathway participates in the effect of HMF on NPC cells, we co-administered an AMPK inhibitor (Compound C) and HMF in two NPC cell lines. As shown in Figures 5A, B, HMF inhibited the proliferation of 5-8F and CNE-2 cells; however, pretreatment with compound C reversed the HMF-induced inhibition of proliferation. Similarly, the relative colony formation was significantly increased in the Compound C/HMF group compared to the group administered HMF alone (Figures 5C–F). Moreover, we also showed that Compound C pretreatment reversed the HMF-induced apoptosis in 5-8F and CNE-2 cells (Figures 5G–J) (*p < 0.05 and **p < 0.01). These results indicated that HMF inhibited the proliferation and induced the apoptosis of NPC cell lines through the AMPK-dependent pathway.

### 3.7 HMF inhibits invasion and migration by regulating the AMPK-dependent signal pathway in NPC cells

Compound C was co-administered with HMF in 5-8F and CNE-2 cells to confirm whether HMF was involved in regulating the invasion and migration of NPC cell lines via the AMPK-related pathway. Compound C was co-administered with HMF in 5-8F and CNE-2 cells. As shown in Figures 5K–N, HMF could inhibit 5-8F and CNE-2 cell invasion, which was ameliorated by pretreatment with Compound C. Similarly, HMF also could inhibit migration of CNE-2 and 5-8F cells, while pretreatment with Compound C reversed this HMF-induced migration inhibition (Figures 5O–I) (*p < 0.05 and **p < 0.01). These results showed that HMF inhibited invasion and migration by activating AMPK-dependent signaling pathways in NPC cells.

### 3.8 HMF inhibits NPC cell growth *in vivo*

**FIGURE 6:**
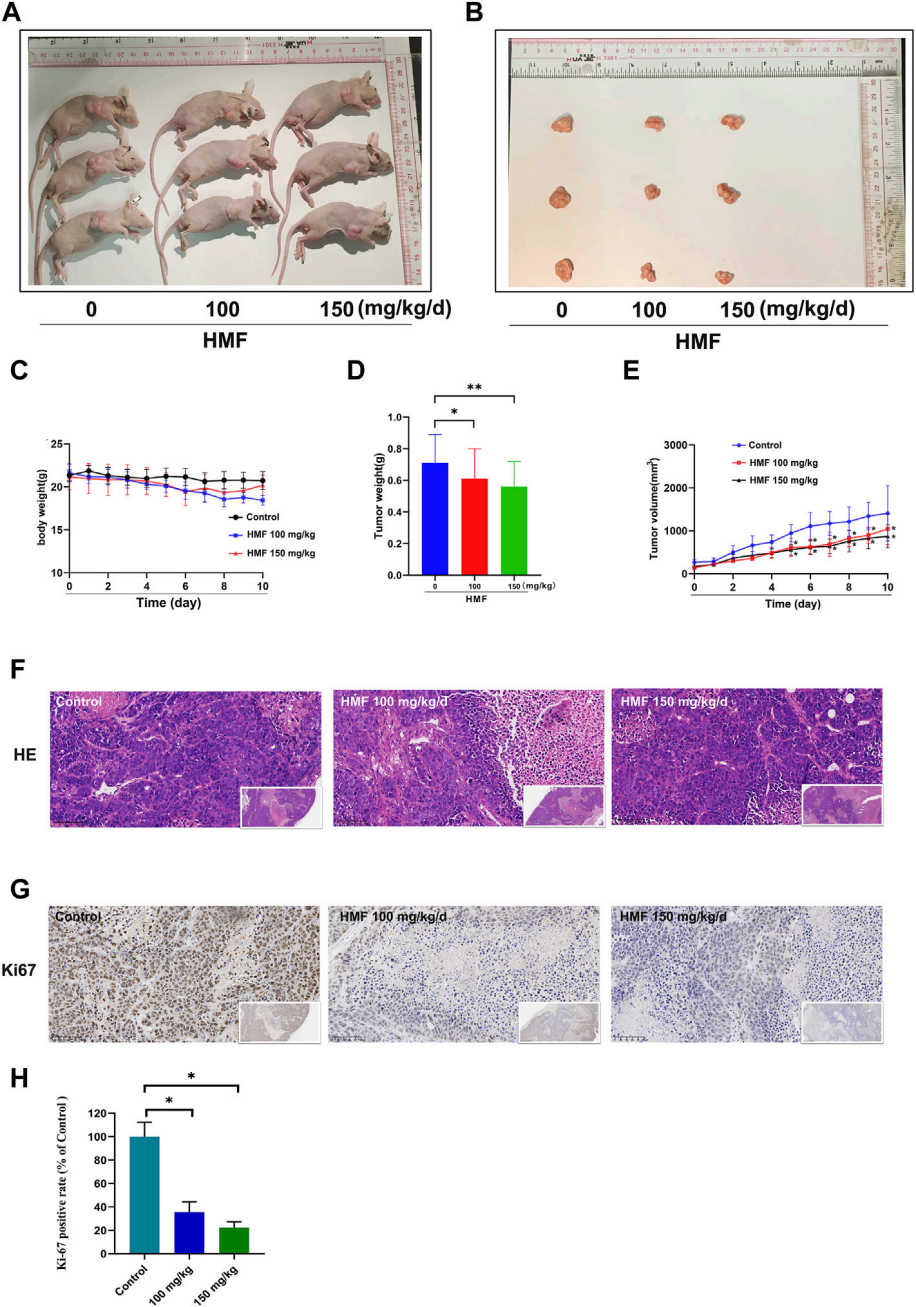
HMF inhibits NPC tumor growth and promotes tumor cell death in nude mice. **(A,B)** NPC tumors of nude mice. **(C)** Daily body weight variation in tumor-bearing nude mice in the control and HMF treatment groups. **(D)** Weight and **(E)** volume changes of implanted tumors for 10 days. **(F)** H&E staining of tumor tissues from each treatment group (400×). **(G)** Ki-67 staining was conducted to detect NPC cell proliferation. **(H)** Quantified analysis of Ki-67 positivity rate (%) and AMPK expression (%). All data are described as the mean ± SD of three independent and repeated experiments. Compared to the control group (0 μM): *p < 0.05; **p < 0.01; ns, not significant.

A CNE-2 cell-transplanted xenograft tumor model in nude mice was performed to assess the anti-tumor action of HMF. The nude mice were split at random into three groups and were administrated HMF (100 and 150 mg/kg/day). After 14 days of implantation and HMF treatment, the tumor volumes and weights were measured after the dissection of the nude mice. As shown in Figures 6A–E, the tumor sizes in the HMF-treated mice were smaller than those in the control mice (*p < 0.05, **p < 0.01); however, no remarkable change in body weight was observed (Figure 6C). H&E staining showed that nude mice administered HMF treatment had worse tumor tissue necrosis than those of the control group. The tumor cells in the control group showed evident nucleolus and large nuclei that were arranged densely. In contrast, a loose arrangement was observed in the HMF-treated groups (Figure 6F). The Ki-67 assay revealed that compared to the control group, HMF treatment inhibited tumor cell proliferation (Figures 6G, H) (*p < 0.05). These results indicated that HMF inhibited NPC cell growth *in vivo*.

## 4 Discussion

HSCCC, unlike the traditional chromatographic column and adsorbent resin, is a continuous liquid–liquid partitioning chromatography with the advantages of a reduced analysis running time, no irreversible adsorption, and a high loading amount. HSCCC has been successfully used for the separation of many natural products due to its flexible two-phase solvent system (Yang et al., 2020; Li et al., 2022). In the present study, four PMFs were successfully isolated and purified from CRCP in large quantities by petroleum ether heated reflux and HSCCC. These compounds were identified as NOB (325 mg), HMF (67 mg), TGN (249 mg), and 5-HPMF (90 mg) by ESI-MS, ^1^H-NMR, and ^13^C-NMR, and all purities were >95.0% by HPLC-PDA method. Hence, HSCCC is an ideal method for efficiently purifying large amounts of PMFs from CRCP. The valorization of PMFs is beneficial to provide a material basis for follow-up research on anti-NPC.

PMFs have been a research focus for their significant and broad pharmacological activities, such as anti-atherosclerosis, anti-inflammatory, and anti-tumor effects (Kong et al., 2020). For instance, TGN inhibits G1/S division and breast cancer cell metastasis by up-regulating p53/P21 and inhibiting matrix metalloproteinase-2 (MMP-2), MMP-9, and vascular endothelial growth factor, respectively (Arivazhagan and Sorimuthu, 2014). We previously reported inhibition of the proliferation and induction of apoptosis in NPC C666-1 cells by nobiletin (Zheng et al., 2019). However, systematic studies on PMF action in NPC are lacking. The present study compared the inhibitory effects of four PMFs (5-HPMF, TGN, NOB, and HMF) on NPC cells by CCK-8 assay. The results showed that the four PMFs inhibited the proliferation of 5-8F and CNE-2 cells in time- and dose-dependent manners. The inhibition effect based on IC_50_ was in the following order: HMF>NOB>TGN>5-HPMF. HMF showed the strongest cytotoxicity, with IC_50_ values of 36.273 ± 0.502 μM (CNE-2) and 31.786 ± 0.841 μM (5-8F). Further studies demonstrated the inhibitory effects of HMF on the proliferation, migration, and invasion and induction of apoptosis in NPC cell lines (CNE-2 and 5-8F). More importantly, HMF (100 and 150 mg/kg/day) could singly inhibit NPC growth *in vivo*.

PMFs attached with more methoxy group show stronger anti-tumor ability since the lower polarity contributes to cancer cell penetration (Gao et al., 2018; Kim and Lim, 2020). 3,5,6,7,8,3′,4′-Heptamethoxyflavone (HMF) contains 2-phenyl chromogenic ketone as the basic parent nucleus and C-3, C-5, C-6, C-7, C-8, C-3′, and C-4′ attached with -OCH_3_, which indicate its lower polarity and planar structure.

Emerging studies have focused on the neuroprotective and antidepressant effect of HMF (Sawamoto et al., 2017). For instance, HMF showed neuroprotection against brain ischemia by inducing BDNF production and anti-inflammatory actions (Okuyama et al., 2014; Okuyama et al., 2015) and demonstrated an immunomodulatory function for the reduction of interleukin-4 expression in CD3/CD28-stimulated spleen cells in mice (Nakajima et al., 2016). However, its anti-tumor activity rarely has been mentioned, and the molecular mechanism for the anti-tumor effects of HMF remains unclear.

AMPK, an evolutionarily highly conserved serine/threonine protein kinase, functions to maintain and regulate the dynamic balance of cell energy by participating in glucose and lipid metabolism, in which it serves as an important physiological and pathological energy receptor in eukaryotic cells. Previous studies demonstrated that AMPK affects tumor cell growth in the following AMPK-dependent pathways. First, AMPK inhibits tumor cell proliferation and protein synthesis by negatively regulating mTOR-dependent signaling pathways. Ribosomal protein S6 kinase, 70 kDa (P70S6K) is one of the main downstream effectors of the AMPK-mTOR signal pathway. In general, mTOR phosphorylates S6K, which in turn phosphorylates the S6 protein of the 40S ribosomal subunit, regulating the initiation of translation, mRNA processing, and cell growth. Malignant tumor cells require large amounts of energy for proliferation. AMPK is activated when energy is depleted, accounting for the inactivation of mTOR and tumor cell growth inhibition. Briefly, AMPK activation regulates tumor cell growth, invasion, and metastasis, whereas the specific action of AMPK on the metastatic potency of NPC cells remains unknown (Zhou et al., 2017). In our study, following HMF treatment, the protein expression levels of p-AMPK, which promotes cell death, and the protein levels of the phosphorylated form of P70S6K and S6 were remarkably inhibited in 5-8F and CNE-2 cells. This observation is consistent with those of previous studies which reported that the AMPK and mTOR pathways might interact in inhibiting the proliferation and inducing the apoptosis of cancer cells, such as colorectal cancer (Dutta et al., 2022), breast cancer (Li et al., 2021), and lung cancer (Song et al., 2021). For instance, the volatile components of frankincense, pine needle, and geranium inhibited the activity, proliferation, migration, and invasion and induced apoptosis of McF-7 human breast cancer cells. In the present study, we found that HMF significantly upregulated AMPK phosphorylation and decreased the protein level of p-P70S6K and S6 in CNE-2 and 5-8F cells, suggesting that HMF induced the activation of an AMPK-mTOR signaling pathway.

AMPK is a critical factor in regulating cellular energy metabolism by promoting ATP production and energy metabolism (Thirupathi and Chang, 2019). The AMPK/COX-2 pathway is also involved in regulating tumor cell growth, migration, and invasion. Cyclooxygenase-2 (COX-2), an inducible central enzyme of prostaglandin biosynthesis, is stimulated by growth factors, inflammatory mediators, and carcinogenic agents; thus, it is also considered an anticancer target, as it is highly expressed in tumor tissues and participates in tumor occurrence and development. MMP-2, which functions to degrade cellular basement membranes, is necessary for malignant tumor cells to penetrate multiple basement membrane barriers around tissues, blood vessels, nerves, and muscles in the process of metastasis. The invasive and metastatic ability of cancer cells occurs due to the metabolites of the cyclooxygenase (COX) and lipoxygenase pathways (PGF2-α and 5-HPETE). Therefore, COX-2 inhibition reduces MMP-2 production, which, in turn, inhibits tumor invasion and metastasis (Reich and Martin, 1996). Highly activated AMPK inhibits the extracellular signal-regulated kinase (ERK) and COX signaling pathways, thereby reducing melanoma metastasis (Kim et al., 2012). Moreover, COX-2 inhibitors affect the apoptosis susceptibility of solid tumors, which is closely related to prognosis. The AMPK/COX-2 signaling pathway is also involved in apoptosis induced by natural products, such as curcumin, quercetin (Lee et al., 2009), and green tea catechin (Park et al., 2009) in colon cancer cells. Our research found that compared to the control group, the HMF treatment group upregulated the p-AMPK/AMPK protein level and downregulated the COX-2 protein level. Combining with the results of Hoechst-33258 staining to detect the level of cell apoptosis, transwell assays to examine cell migration, and wound scratch healing assays to measure invasion ability, HMF may inhibit NPC cell growth through the AMPK/COX-2 signaling pathway.

Previous studies demonstrated that AMPK induced apoptosis by regulating p53-dependent signaling pathways. p53, the most commonly mutated gene in cancer, is considered a tumor suppressor under metabolic stress. Deletion or mutation of p53 leads to uncontrolled cell growth, apoptosis inhibition, chemotherapy resistance, and promotion of tumor formation (Liu et al., 2011). When energy is scarce in the body, AMPK phosphorylates p53 to limit cell growth and save energy. Hence, increased AMPK activity promotes p53 transcription and inhibits cancer cell growth rates. Hemistepsin A could inhibit HCC cell proliferation and induce G0/G1 cell cycle arrest and mitochondrial-related apoptosis by activating AMPK/p53 pathways in the human hepatoma Huh7 cell line (Baek et al., 2020). Analogously, α-lipoic acid inhibits cell proliferation, adhesion, invasion, and colony formation in the human HT29 and mouse MCA38 colon cancer cell lines via the AMPK/p53 signaling pathway (Park et al., 2015). In the present study, after HMF treatment, the expression levels of p-p53/p53 and p-AMPK/AMPK on CNE-2 and 5-8F in the HMF group were significantly increased compared to those in the control group. Synthesizing the aforementioned results of apoptosis indicators, we concluded that HMF might mediate the AMPK/p53 signaling pathway to regulate apoptosis and exert an anti-NPC effect.

While multiple studies have reported that the upregulation of AMPK could inhibit cancer cell growth, few studies have focused on the inhibitory effect of AMPK-activated drugs on NPC cells (Xia et al., 2021; Liu et al., 2022). Previous studies reported that metformin and some plant-derived products, including curcumol, lupeol, resveratrol, and andrographolide, inhibited NPC cell growth by AMPK activation. Metformin, a synthetic biguanide, is used for the treatment of type II diabetes and induces both AMPK-dependent and AMPK-independent genes/pathways, accounting for its inhibitory effects on cancer cell growth (An and He, 2016; Safe et al., 2018). For instance, metformin inhibited C666-1 cell proliferation by decreasing cyclin D1 levels and inducing G1 cell cycle arrest, which may be involved in the AMPK-mediated inhibition of mTORC1 signaling (Zhao et al., 2011). Among the aforementioned natural AMPK-activated drugs, curcumol, lupeol, and andrographolide are terpenoid compounds with low polarity. Curcumol-induced autophagy and apoptosis through the activation of the AMPK/mTOR pathway in CNE-2 cells (Wang et al., 2021). Analogously, andrographolide suppressed the proliferation and induced the apoptosis of C666-1 cells through regulation of the LKB1/AMPK/mTOR signal pathway (Wu et al., 2018). Lupeol modulated apoptosis, ferroptosis, and inflammation in CNE1 nasopharyngeal carcinoma cells through the AMPK/NF-κB pathway (Zhou et al., 2022). Resveratrol, a natural non-flavonoid phenolic compound found in red grapes, inhibited proliferation and induced apoptosis in the C666-1 nasopharyngeal carcinoma cell line via AMPK activation (Cai et al., 2015). PMFs attached with more methoxy group show stronger anti-tumor effects since their lower polarity contributes to the penetration of cancer cells. In our study, HMF belonged to PMFs attached with seven methoxy groups, indicating its low polarity and plan structure. HMF-induced AMPK activation inhibited the growth, invasion, and metastatic potency of NPC cells by downregulating mTOR signaling pathway activation and COX-2 protein levels, as well as enhancing p53 phosphorylation.

**FIGURE 7:**
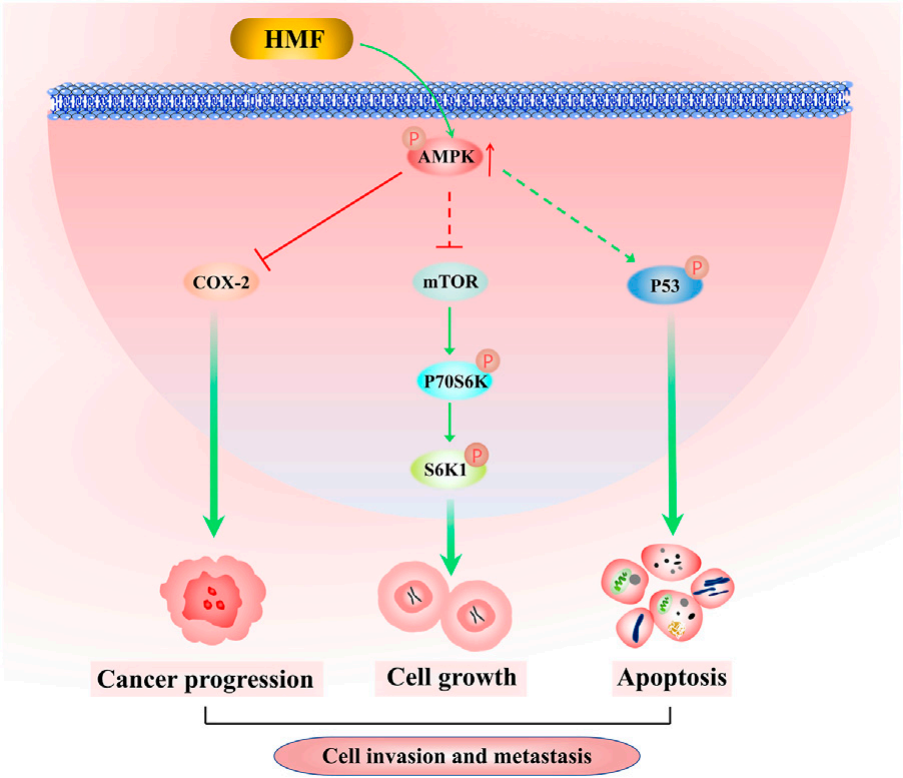
Molecular mechanism by which HFM inhibits nasopharyngeal carcinoma cell growth. HMF-induced AMPK activation inhibits NPC cell growth, invasion, and metastatic potency via downregulation of the activation of the mTOR signaling pathway and COX-2 protein levels, as well as enhancement of p53 phosphorylation.

In conclusion, the results of this study demonstrated for the first time that HMF purified from CRCP significantly inhibited the proliferation and induced apoptosis of NPC cells (CNE-2 and 5-8F) and also inhibited NPC cell migration and invasion. The results of the tumor transplantation experiment in nude mice confirmed the inhibitory effect of HMF on NPC cell growth *in vivo*. Further investigation revealed that HMF appeared to act via AMPK-dependent signaling pathways involved in NPC cell growth, migration, and invasion inhibition (Figure 7). These findings provide a preliminary experimental basis for treating NPC and the development and utilization of PMFs in CRCP.

## Data Availability

The original contributions presented in the study are included in the article/Supplementary Material. Further inquiries can be directed to the corresponding authors.

## Abbreviations

AMPK, AMP-activated protein kinase; CRCP, *Citrus reticulata* ‘Chachi’ pericarp; FBS, fetal bovine serum; HSCCC, high-speed counter-current chromatography; HMF, 3,5,6,7,8,3′,4′- heptamethoxyflavone; NOB, nobiletin; NPC, nasopharyngeal carcinoma; PBS, phosphate-buffered saline; IMRT, intensity-modulated radiotherapy; PMFs, polymethoxyflavonoids; PE, petroleum ether; TGN, tangeretin; 5-HPMF, 5-hydroxy-6,7,8,3′,4′-pentamethoxyflavone.
